# Integrating Structural and Metabolic Neuroimaging Biomarkers for Alzheimer’s Disease Diagnosis and Cognitive Score Estimation via Cross-Modal Gated Learning

**DOI:** 10.3390/biology15131091

**Published:** 2026-07-07

**Authors:** Chenyu Tang, Lin Shi, Shoukun Xu

**Affiliations:** 1 Wang Zheng School of Microelectronics, Changzhou University, Changzhou 213164, China; s24060854026@smail.cczu.edu.cn; 2 School of Computer Science and Artificial Intelligence, Changzhou University, Changzhou 213164, China; slcczu@cczu.edu.cn

**Keywords:** Alzheimer’s disease, structural MRI, FDG-PET, multimodal neuroimaging, cognitive score estimation, cross-modal gated learning, multi-task learning, HSIC

## Abstract

Structural atrophy and metabolic dysfunction provide complementary biomarkers for Alzheimer’s disease (AD), and their joint modeling may support diagnostic assessment and cognitive score estimation. However, many multimodal methods rely on global fusion and insufficiently enhance cross-modal consistency before interaction, limiting the discriminative quality and clinical relevance of learned representations. We propose CGMF-Net, a cross-modal gated learning framework for joint AD classification and clinical score estimation using paired structural MRI (sMRI) and fluorodeoxyglucose PET (FDG-PET) data. CGMF-Net extracts multi-scale representations from both modalities, introduces a Cross-Modal Similarity Gate to strengthen consistent structural–metabolic responses before fusion, and employs bi-directional cross-attention to capture complementary interactions. The shared representation is optimized with classification supervision, MMSE-based auxiliary regression, and HSIC regularization to improve discriminability and reduce redundant coupling between directional representations. Experiments on ADNI demonstrate that CGMF-Net achieves the best overall classification performance among the compared methods, with 94.22% ACC and 97.74% AUC for AD vs. CN, and 86.67% ACC and 94.84% AUC for AD vs. MCI, while also showing favorable ADNI-2 to ADNI-1 generalization and competitive estimation of ADAS13, CDRSB, and MMSE. These results suggest that cross-modal gated learning provides clinically relevant multimodal representations for AD diagnosis and cognitive score estimation.

## Simple Summary

Alzheimer’s disease is associated with structural brain atrophy and metabolic dysfunction, which can be observed using structural magnetic resonance imaging (sMRI) and fluorodeoxyglucose positron emission tomography (FDG-PET). However, effectively integrating these complementary neuroimaging biomarkers remains challenging. In this study, we propose CGMF-Net, a cross-modal gated learning framework for Alzheimer’s disease classification and cognitive score estimation. The model enhances consistent structural and metabolic responses before fusion and further models complementary interactions between the two modalities. Experiments on ADNI data show that CGMF-Net improves AD classification, generalizes favorably from ADNI-2 to ADNI-1, and estimates clinically relevant cognitive scores. These findings suggest that integrating structural and metabolic biomarkers through cross-modal gated learning may support computer-aided assessment of Alzheimer’s disease.

## 1. Introduction

Alzheimer’s disease (AD) is one of the most prevalent neurodegenerative disorders and has become a major public health challenge worldwide, placing an increasing burden on patients, families, and health-care systems [1]. AD is characterized by progressive brain atrophy, metabolic dysfunction, and cognitive decline, making neuroimaging an important tool for characterizing disease-related brain changes and supporting computer-aided diagnostic assessment [2,3]. Among commonly used neuroimaging modalities, structural magnetic resonance imaging (sMRI) provides anatomical information related to cortical and subcortical atrophy, whereas fluorodeoxyglucose positron emission tomography (FDG-PET) reflects cerebral glucose metabolism and metabolic abnormalities. Therefore, FDG-PET was selected in this study to characterize metabolic dysfunction complementary to sMRI-derived structural atrophy, rather than to replace amyloid PET for molecular amyloid assessment. These two modalities provide complementary structural and metabolic neuroimaging biomarkers for AD analysis. Therefore, jointly modeling sMRI and FDG-PET is valuable not only for distinguishing diagnostic groups but also for capturing imaging patterns associated with cognitive status and clinical assessment [4,5].

Driven by recent advances in deep learning, multimodal neuroimaging has become an important direction for AD classification, as it can better characterize disease-related alterations than single-modality analysis [6,7,8]. Existing multimodal AD methods mainly differ in their fusion strategies. Early studies often adopted relatively simple feature combination schemes, whereas recent deep models have explored more expressive forms of multimodal interaction, including adversarial feature fusion, hypergraph-based relationship modeling, disease-induced joint learning, hierarchical attention, and multiscale cross-modal enhancement [5,9,10,11,12,13]. Although these methods have achieved promising performance, two limitations remain insufficiently addressed in paired sMRI–FDG-PET analysis. First, many existing methods mainly focus on global fusion or late-stage aggregation, while cross-modal consistency across different semantic levels is not explicitly enhanced before fusion. As a result, inconsistent or redundant responses from paired modalities may be propagated into the fusion stage, weakening the quality of the learned multimodal representation. Second, cross-modal interactions are often modeled in a relatively global or insufficiently directional manner, which may limit the ability to fully capture complementary structure-guided metabolic patterns and metabolism-guided structural patterns. These limitations motivate the development of a multimodal framework that can strengthen structural–metabolic consistency before fusion and explicitly model complementary interactions between sMRI and FDG-PET.

In addition to AD classification, cognitive score estimation provides complementary information for characterizing disease severity and cognitive status. Multi-task learning offers an effective way to improve generalization by enabling related tasks to share informative representations [14]. In AD analysis, categorical diagnostic labels and continuous cognitive measures are naturally correlated, since both reflect the underlying disease status from different perspectives. Several studies have explored joint modeling of disease classification and clinical score regression. For example, Zhang and Shen [4] introduced an early multimodal multi-task framework for jointly predicting regression and classification variables in AD. Liu et al. [15] proposed a deep multi-task multi-channel model that unifies classification and score-related prediction. Lian et al. [16] developed a multi-task weakly supervised attention network for dementia status estimation from sMRI, while El-Sappagh et al. [17] extended multi-task learning to multimodal disease progression analysis. More recent studies have also shown that deep models can capture meaningful relationships between neuroimaging features and cognitive measures [18,19]. Nevertheless, existing multi-task methods remain limited for our target setting. Some approaches are based on a single modality and therefore cannot fully exploit the complementary information from paired sMRI and FDG-PET. Some multimodal studies emphasize disease progression modeling rather than joint AD classification and cognitive score estimation under a paired baseline imaging setting. Moreover, when shared representations are derived from cross-modal interaction, the relationship between different fusion directions is rarely explicitly regularized. Therefore, a unified multimodal multi-task framework is still needed to jointly perform AD classification and cognitive score estimation while improving the quality of shared structural–metabolic representations.

To address these limitations, we propose a Cross-Modal Gated Multi-task Fusion Network (CGMF-Net) for joint AD classification and cognitive score estimation from paired sMRI and FDG-PET data. The proposed framework integrates multi-scale feature extraction, Cross-Modal Similarity Gate (CMSGate), bi-directional cross-attention, and multi-task supervision within a unified end-to-end architecture. Specifically, multi-scale features are extracted from both modalities to preserve complementary structural and metabolic information. CMSGate is introduced before explicit multimodal fusion to enhance cross-modal consistency across multiple semantic levels. Bi-directional cross-attention is then used to model complementary interactions in the MRI-to-PET and PET-to-MRI directions. The resulting shared representation is jointly optimized for AD classification and cognitive score regression, and Hilbert–Schmidt Independence Criterion (HSIC) regularization is further incorporated to reduce redundant coupling between directional representations. In the current implementation, the Mini-Mental State Examination (MMSE) score is used as the auxiliary regression target, while the overall framework is designed to support joint modeling of multiple clinically relevant cognitive scores, such as ADAS13, CDRSB, and MMSE.

The main contributions of this work are summarized as follows:

- We propose CGMF-Net, a cross-modal gated multi-task learning framework for joint AD classification and cognitive score estimation from paired sMRI and FDG-PET data. By jointly optimizing disease classification and clinical score regression within a shared architecture, the proposed framework links structural and metabolic neuroimaging biomarkers with cognitive status, improving the clinical relevance and generalizability of the learned multimodal representation.
- We introduce a Cross-Modal Similarity Gate (CMSGate) to enhance structural–metabolic consistency before explicit multimodal fusion. CMSGate adaptively strengthens responses that are consistently supported by the complementary modality across multiple semantic levels, helping suppress inconsistent or redundant signals and providing more informative representations for subsequent cross-modal interaction.
- We design a bi-directional cross-attention strategy with joint supervision from disease labels, cognitive scores, and HSIC regularization. This design explicitly models complementary interactions in both MRI-to-PET and PET-to-MRI directions, while reducing redundant coupling between directional representations. As a result, the learned representation becomes more discriminative and interpretable for AD classification and cognitive score estimation.

## 2. Materials and Methods

### 2.1. Dataset and Preprocessing

The data used in this study were obtained from the Alzheimer’s Disease Neuroimaging Initiative (ADNI) database, including the ADNI-1 and ADNI-2 cohorts. Subjects were categorized into three diagnostic groups: Alzheimer’s disease (AD), mild cognitive impairment (MCI), and cognitively normal controls (CN). Only subjects with paired structural magnetic resonance imaging (sMRI) and fluorodeoxyglucose positron emission tomography (FDG-PET) data were included for multimodal analysis. As summarized in Table 1, the ADNI-1 cohort contains 360 subjects, including 84 AD, 183 MCI, and 93 CN cases, while the ADNI-2 cohort contains 709 subjects, including 136 AD, 328 MCI, and 245 CN cases. In total, 1069 subjects were included in this study, comprising 220 AD, 511 MCI, and 338 CN cases.

In addition to diagnostic labels, clinically relevant cognitive scores were used to evaluate whether the learned multimodal representation could capture continuous disease-related variations. Specifically, ADAS13, CDRSB, and MMSE were considered for clinical score regression evaluation. In the proposed multi-task framework, MMSE was further used as a classification-oriented auxiliary regression target to provide cognitive-status-related supervision, and its contribution was analyzed in the ablation study.

For neuroimaging preprocessing, we followed a unified multimodal pipeline for sMRI and FDG-PET. First, MRI scans were skull-stripped to remove non-brain tissues. Each FDG-PET scan was then linearly registered to its paired MRI scan to ensure subject-level cross-modal alignment. Subsequently, each MRI scan was affinely registered to the standard MNI template, and the same spatial transformation was applied to the corresponding FDG-PET scan to preserve anatomical correspondence between the two modalities. After registration, both modalities were resampled into a unified MNI-conformant space. During network training, all input volumes were resized to a fixed resolution of 112 × 128 × 112 to match the network architecture and reduce computational cost. Finally, voxel intensities were normalized independently for each modality using per-volume z-score normalization. No additional multi-site harmonization method, such as ComBat or cohort-level intensity matching, was applied in this study. Instead, a unified preprocessing pipeline and per-volume z-score normalization were used for all cohorts, and cross-dataset generalization from ADNI-2 to ADNI-1 was further evaluated to assess robustness under cohort-level distribution differences.

### 2.2. Overall Framework

**Figure 1:**
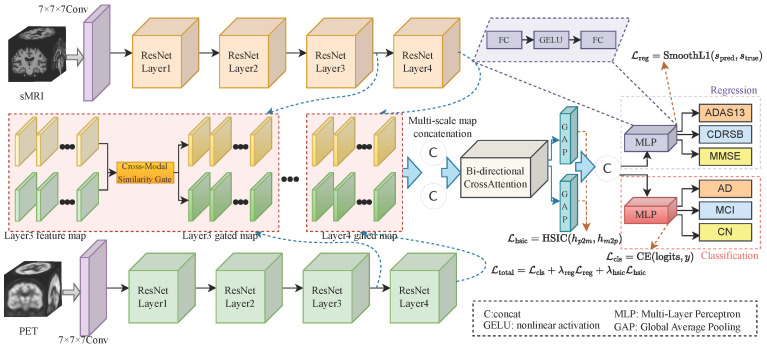
Overall architecture of the proposed Cross-Modal Gated Multi-task Fusion Network (CGMF-Net).

Given paired sMRI and FDG-PET volumetric data, we propose the Cross-modal Gated Multi-task Fusion Network (CGMF-Net), which jointly performs disease classification and clinical score regression within a unified cross-modal learning framework. As illustrated in Figure 1, the network comprises two modality-specific 3D branches, each taking MRI or PET as input. Each branch is built upon a 3D residual backbone to extract hierarchical representations from volumetric neuroimaging data [20]. Rather than relying solely on the deepest features, we retain selected intermediate representations from the backbone to preserve richer semantic information at complementary scales. This design provides more informative inputs for subsequent cross-modal interaction and fusion, enabling the model to better capture structural and metabolic patterns from paired neuroimaging data.

Specifically, for each modality, we instantiate the above design by selecting the features from layer3 and layer4 as the retained intermediate representations, since these two stages provide a suitable balance between semantic abstraction and multi-scale complementarity. Here, “semantic levels” refer to different hierarchical feature levels learned by the 3D-ResNet18 backbone, specifically the layer3 and layer4 representations, rather than handcrafted visual primitives, predefined anatomical-region groupings, or clinical semantic categories. The 3D-ResNet18 backbone consists of an initial 7 × 7 × 7 convolution followed by four residual stages and was selected as a moderate-depth architecture to balance volumetric representation capacity and overfitting risk. Since sMRI and FDG-PET were registered to the same MNI-conformant space and resized to the same input resolution, the two modality-specific branches operate on spatially aligned voxel grids. Under this setting, the progressively enlarged receptive fields of layer3 and layer4 provide region-level contextual representations, which are suitable for capturing structural atrophy patterns in sMRI and metabolic hypometabolism patterns in FDG-PET, while reducing sensitivity to isolated voxel-level noise. The selected features are first projected into a unified representation space and then enhanced by the proposed Cross-Modal Similarity Gate (CMSGate) before cross-modal fusion. The gated multi-scale features within the same modality are subsequently concatenated to form compact modality-aware representations. On this basis, we further introduce a bi-directional cross-attention mechanism to explicitly model MRI-to-PET and PET-to-MRI interactions, thereby capturing complementary dependencies between the two modalities from both directions [21]. The resulting bi-directional features are aggregated into a shared representation, which is then used for both disease classification and clinical score regression. In addition, MMSE is further incorporated as an auxiliary supervision signal for classification. In this manner, multi-scale feature extraction, cross-modal gated enhancement, bi-directional attention fusion, clinical score regression, and task-related supervision are seamlessly integrated into an end-to-end trainable framework.

### 2.3. Cross-Modal Similarity Gate

To emphasize response regions with consistent discriminative relevance across modalities prior to explicit cross-modal fusion, we design the Cross-Modal Similarity Gate (CMSGate) module, which is applied independently to the retained features from layer3 and layer4. As discussed above, these two stages are selected because they provide a suitable trade-off between semantic abstraction and multi-scale complementarity: compared with shallower layers, they contain more discriminative semantic information for modeling cross-modal consistency, while compared with relying only on the deepest layer, their joint use helps preserve complementary cues from high-level semantics and local spatial details. Based on this design, CMSGate leverages similarity constraints from the complementary modality to adaptively enhance responses with higher cross-modal consistency, thereby highlighting disease-relevant shared information while suppressing inconsistent or redundant signals.

Formally, let the input features at a given scale for MRI and PET be denoted as $X^{M}$ and $X^{P}$. To reduce inter-modality distribution differences and provide a unified representation for subsequent similarity computation, we first normalize both modalities using Layer Normalization:

(1) $$\left\{\begin{array}{cc} {\hat{X}}^{M} & =\mathrm{LN}(X^{M}),\\ {\hat{X}}^{P} & =\mathrm{LN}(X^{P}), \end{array}\right.$$

where LN(·) denotes Layer Normalization. This maps MRI and PET features into a comparable representation space for cross-modal similarity modeling.

Next, we compute bidirectional cross-modal similarity to characterize response consistency between modalities at different spatial positions:

(2) $$\left\{\begin{array}{cc} S_{M\to P} & =\frac{{\hat{X}}^{M}{\left({\hat{X}}^{P}\right)}^{⊤}}{\sqrt{d}},\\ S_{P\to M} & =\frac{{\hat{X}}^{P}{\left({\hat{X}}^{M}\right)}^{⊤}}{\sqrt{d}}, \end{array}\right.$$

where *d* is the feature dimension, and $S_{M\to P}$ and $S_{P\to M}$ quantify the strength of cross-modal correlation in each direction. This allows the module to explicitly measure how well local responses in one modality match positions in the other.

To extract the strongest response at each spatial position, we perform max-pooling along the corresponding dimension:

(3) $$\left\{\begin{array}{cc} g_{i}^{M} & =\max_{j}S_{M\to P}\left(i,j\right),\;i=1,\ldots ,L,\\ g_{j}^{P} & =\max_{i}S_{P\to M}\left(i,j\right),\;j=1,\ldots ,L, \end{array}\right.$$

where *L* denotes the number of spatial positions. The resulting vectors $g^{M}$ and $g^{P}$ serve as position-wise gating cues, compressing the similarity matrix into an importance map for adaptive enhancement.

To enhance regions with strong cross-modal consistency while preserving the original discriminative information, we adopt a residual gating strategy:

(4) $$\left\{\begin{array}{cc} {\tilde{X}}^{M} & =\left(1+\gamma \;\sigma (g^{M})\right)⊙X^{M},\\ {\tilde{X}}^{P} & =\left(1+\gamma \;\sigma (g^{P})\right)⊙X^{P}, \end{array}\right.$$

where σ(·) is the Sigmoid function, γ is a learnable scaling parameter, and ⊙ denotes element-wise multiplication with broadcasting along the channel dimension. This residual gating enhances high-consistency regions without overwriting the original features, preserving structural and metabolic information while emphasizing discriminative shared representations.

**Figure 2:**
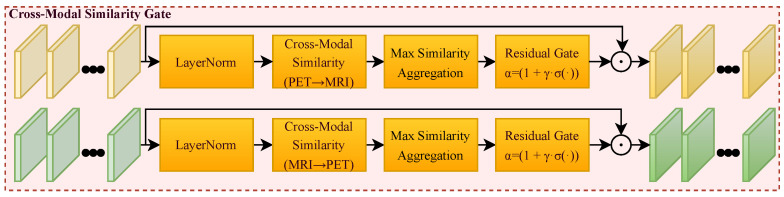
Illustration of the proposed Cross-Modal Similarity Gate (CMSGate). At each scale, the module first applies Layer Normalization, then computes bidirectional cross-modal similarity, performs max-similarity aggregation to obtain position-wise gating cues, and finally enhances the input maps via a residual gate.

The CMSGate workflow is illustrated in Figure 2. Notably, independent CMSGate modules are used for layer3 and layer4, enabling each semantic scale to learn a modality-specific gating pattern for more informative inputs to the subsequent bi-directional cross-attention fusion.

### 2.4. Bi-Directional Cross-Attention

After CMSGate enhancement, the MRI and PET features at the layer3 and layer4 scales contain more salient cross-modal consistent responses. To further model the complementary dependencies between the two modalities, we first concatenate the multi-scale features within each modality and then introduce a bi-directional Cross-Attention mechanism to explicitly perform cross-modal information interaction from both directions. This design is built upon the standard scaled dot-product attention formulation [21].

Specifically, let the CMSGate-enhanced multi-scale features of MRI and PET be denoted as ${\tilde{X}}_{l3}^{M}$, ${\tilde{X}}_{l4}^{M}$, ${\tilde{X}}_{l3}^{P}$, and ${\tilde{X}}_{l4}^{P}$, respectively. We first concatenate the two scales within each modality to obtain compact representations for subsequent cross-modal interaction:

(5) $$\left\{\begin{array}{cc} X_{c}^{M} & =\mathrm{Concat}\;\left({\tilde{X}}_{l3}^{M},{\tilde{X}}_{l4}^{M}\right),\\ X_{c}^{P} & =\mathrm{Concat}\;\left({\tilde{X}}_{l3}^{P},{\tilde{X}}_{l4}^{P}\right), \end{array}\right.$$

where $X_{c}^{M}$ and $X_{c}^{P}$ denote the multi-scale fused features of MRI and PET, respectively. Through this operation, discriminative information from different semantic levels is integrated into a unified representation, providing more informative inputs for subsequent cross-modal attention.

In cross-modal fusion, we adopt the standard scaled dot-product attention, defined as

(6) $$\mathrm{Attn}\left(Q,K,V\right)=\mathrm{Softmax}\left(\frac{QK^{⊤}}{\sqrt{d}}\right)V,$$

where Q, K, and V denote the query, key, and value, respectively, and *d* is the feature dimension. This mechanism adaptively aggregates task-relevant information in the value features according to the correlation between the query and the key [21].

Based on this formulation, we further construct a bi-directional Cross-Attention module to explicitly model the complementary interactions between MRI and PET in both directions:

(7) $$\left\{\begin{array}{cc} Z_{P\to M} & =\mathrm{Attn}\;\left(\mathrm{LN}\left(X_{c}^{M}\right),X_{c}^{P},X_{c}^{P}\right),\\ Z_{M\to P} & =\mathrm{Attn}\;\left(\mathrm{LN}\left(X_{c}^{P}\right),X_{c}^{M},X_{c}^{M}\right), \end{array}\right.$$

where $Z_{P\to M}$ denotes the cross-modal fused feature obtained by using MRI as the query and PET as the key and value, while $Z_{M\to P}$ denotes the fused feature obtained by using PET as the query and MRI as the key and value. Layer Normalization is applied only to the query branch to improve representation stability during cross-modal interaction.

**Figure 3:**
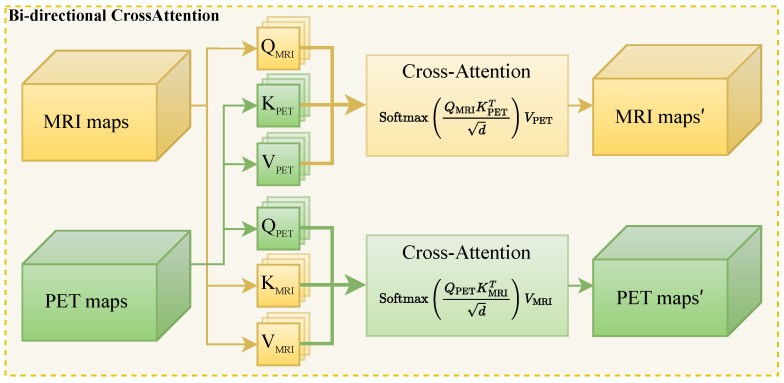
Illustration of the bi-directional cross-attention mechanism. One direction uses MRI features as queries and PET features as keys and values, while the other direction uses PET features as queries and MRI features as keys and values, enabling explicit bidirectional cross-modal interaction.

The overall interaction process of the bi-directional Cross-Attention module is illustrated in Figure 3.

Through this bi-directional design, the model can not only select metabolic information from PET that is most relevant to the current MRI responses but also identify structural information from MRI that is most relevant to the current PET responses. Therefore, the two interaction directions characterize two complementary relationships, namely structure-guided metabolism and metabolism-guided structure, which facilitate more sufficient mining of AD-related multimodal discriminative patterns. The resulting fused features from both directions are further aggregated into direction-level representations, which serve as the basis for constructing the subsequent shared representation.

### 2.5. Multi-Task Prediction and Optimization

After the bi-directional cross-attention module, the network obtains cross-modal fused features from two complementary interaction directions. To obtain compact representations for downstream prediction, global average pooling (GAP) is applied to the fused features from both directions:

(8) $$\left\{\begin{array}{cc} h_{P\to M} & =\mathrm{GAP}\;\left(Z_{P\to M}\right)=\frac{1}{L}\sum_{i=1}^{L}Z_{P\to M}\left(i,:\right),\\ h_{M\to P} & =\mathrm{GAP}\;\left(Z_{M\to P}\right)=\frac{1}{L}\sum_{i=1}^{L}Z_{M\to P}\left(i,:\right), \end{array}\right.$$

where $h_{P\to M}$ and $h_{M\to P}$ denote the direction-level representations aggregated from the PET-to-MRI and MRI-to-PET interaction branches, respectively, and *L* is the number of spatial positions.

The final shared representation is constructed by concatenating the two direction-level representations:

(9) $$f=\mathrm{Concat}\;\left(h_{P\to M},h_{M\to P}\right),$$

where f preserves complementary information from both interaction directions and serves as the input for multi-task prediction.

Based on f, two lightweight task-specific prediction heads are used for disease classification and clinical score regression:

(10) $$\left\{\begin{array}{cc} \ell_{\mathrm{cls}} & ={\mathrm{MLP}}_{\mathrm{classification}}\;\left(f\right),\\ s_{\mathrm{pred}} & ={\mathrm{MLP}}_{\mathrm{regression}}\;\left(f\right), \end{array}\right.$$

where $\ell_{\mathrm{cls}}$ denotes the classification logits and $s_{\mathrm{pred}}$ denotes the predicted clinical score. The regression head is formulated for a representative clinical score for notational simplicity, and the same formulation can be instantiated for different clinically relevant scores. By jointly modeling diagnostic labels and continuous cognitive measures, the shared representation is encouraged to encode both class-discriminative and disease-severity-related information [4,13,14,17].

For disease classification, cross-entropy loss is adopted:

(11) $$L_{\mathrm{cls}}=\mathrm{CE}\;\left(\ell_{\mathrm{cls}},y\right),$$

where *y* is the ground-truth diagnostic label. For clinical score regression, SmoothL1 loss is used to reduce the influence of outliers while maintaining stable optimization:

(12) $$L_{\mathrm{reg}}=\mathrm{SmoothL}1\;\left(s_{\mathrm{pred}},s_{\mathrm{true}}\right),$$

where $s_{\mathrm{true}}$ denotes the ground-truth clinical score [22].

In addition, HSIC regularization is imposed on the two direction-level representations:

(13) $$L_{\mathrm{hsic}}=\mathrm{HSIC}\;\left(h_{P\to M},h_{M\to P}\right),$$

where HSIC measures statistical dependence between two representations [23]. In our implementation, HSIC was computed using an RBF kernel. The kernel bandwidth was not manually fixed; instead, it was adaptively determined by the median heuristic using the non-zero pairwise squared distances within each mini-batch. This term reduces redundant coupling between the two interaction directions before shared fusion and encourages the model to preserve complementary cross-modal information.

The overall objective is defined as:

(14) $$L_{\mathrm{total}}=L_{\mathrm{cls}}+\lambda_{\mathrm{reg}}L_{\mathrm{reg}}+\lambda_{\mathrm{hsic}}L_{\mathrm{hsic}},$$

where $\lambda_{\mathrm{reg}}$ and $\lambda_{\mathrm{hsic}}$ control the contributions of the regression loss and HSIC regularization, respectively. In the classification-oriented training setting, MMSE is used as the auxiliary regression target, and its effect is further evaluated in the ablation study. We acknowledge that MMSE is closely related to cognitive impairment and may be associated with the diagnostic grouping process in ADNI. Therefore, the MMSE-based auxiliary regression branch should be interpreted as cognitive-status-related and potentially label-proximal supervision rather than as an independent diagnostic biomarker. Importantly, MMSE was used only as an auxiliary regression target during training; it was not provided as an input feature to the model, and the predicted MMSE score was not directly used by the classification head. Although the classification and clinical score regression tasks are jointly optimized, the predicted clinical score is not directly used as an input to the classification head. Instead, the regression loss provides soft auxiliary supervision through the shared representation, encouraging the model to learn disease-severity-related features associated with diagnostic status rather than enforcing a deterministic output-level consistency between the two predictions. In addition, the direction of disease severity depends on the specific clinical scale; for example, higher ADAS13/CDRSB scores and lower MMSE scores generally indicate more severe impairment.

### 2.6. Experimental Settings

All disease classification experiments were conducted on the ADNI-2 cohort using five-fold cross-validation at the subject level. Three binary classification tasks were evaluated: AD vs. CN, AD vs. MCI, and CN vs. MCI. The data partition was performed at the subject level to ensure that samples from the same subject did not appear in different folds. No additional class-imbalance handling strategy, such as over-sampling, under-sampling, or class-weighted loss, was applied during training. The classification branch was optimized using the standard cross-entropy loss. To provide a more balanced evaluation under potentially imbalanced class distributions, we reported multiple classification metrics, including ACC, SEN, SPEC, and AUC, rather than relying solely on accuracy. For each classification task, the proposed model was trained using paired sMRI and FDG-PET data, and the shared multimodal representation was optimized by classification loss, auxiliary clinical score regression loss, and HSIC regularization.

Clinical score regression experiments were also conducted on the ADNI-2 cohort to evaluate the ability of the learned multimodal representation to capture continuous cognitive and disease-severity-related variations. Three clinically relevant cognitive scores were considered, including ADAS13, CDRSB, and MMSE. In the classification-oriented multi-task training, MMSE was used as the auxiliary regression target because it directly reflects global cognitive status and provides complementary supervision to the diagnostic labels. The regression performance on ADAS13, CDRSB, and MMSE was reported separately to further assess the clinical relevance of the learned representation.

To evaluate the generalization ability of the proposed model under cohort-level distribution differences, cross-dataset experiments were conducted by training the model on ADNI-2 and testing it on ADNI-1. The ADNI-1 cohort was used only as the target test set and was not involved in model training or model selection. This setting was used for both disease classification and clinical score regression, providing a more practical evaluation of cross-cohort robustness. The held-out folds in ADNI-2 and the ADNI-1 external cohort were used only for final evaluation. Hyperparameter selection and parameter sensitivity analyses were based on the training/validation procedure, without using test-fold or external-test performance for model selection.

All experiments were conducted under the same training and evaluation protocol for fair comparison. To clarify the input settings of the comparison methods, all compared models were evaluated using the same subject-level data splits, preprocessing pipeline, and paired sMRI and FDG-PET inputs whenever applicable. The multimodal comparison methods used the two modalities according to their original fusion designs, while the ResNet18 backbone baseline was implemented under the same paired-input setting for consistency. All baseline results reported in this study were obtained from our reimplementation and evaluation under this unified protocol, rather than directly taken from the original publications. For implementation details, each modality-specific branch used a 3D-ResNet18 backbone; the layer3 and layer4 outputs had 256 and 512 channels, respectively, and were projected into 512-dimensional token representations before entering CMSGate. All 3D backbones were trained from scratch without external pre-trained weights, and overfitting was mitigated by using a moderate-depth 3D-ResNet18 backbone, subject-level cross-validation, multi-task auxiliary supervision, HSIC regularization, and cross-dataset generalization evaluation. Each model was trained for 40 epochs with a batch size of 16 using the Adam optimizer, with an initial learning rate of $\mathrm{LR}=10^{-4}$. To statistically validate the main classification comparison, paired *t*-tests were performed on fold-wise AUC values between CGMF-Net and the best competing baseline for each classification task. The loss weights were set to reg=0.2 and hsic=0.05, according to the parameter sensitivity analysis in Section 3.5; no dynamic loss weighting strategy, such as GradNorm or Kendall’s homoscedastic uncertainty weighting, was used. The same hyperparameter settings were used across the classification tasks unless otherwise specified.

For classification tasks, we report Accuracy (ACC), Sensitivity (SEN), Specificity (SPEC), and Area Under the Receiver Operating Characteristic Curve (AUC). For clinical score regression, Pearson correlation coefficient (CC) and Root Mean Squared Error (RMSE) were used as evaluation metrics. Unless otherwise specified, all quantitative results are reported as mean ± standard deviation. To further assess statistical significance, paired *t*-tests were performed on fold-wise AUC values between CGMF-Net and the best competing baseline for each classification task.

## 3. Results

### 3.1. Classification Performance

We evaluate the proposed model on three binary classification tasks using the ADNI-2 cohort, with all experiments conducted under five-fold cross-validation at the subject level. Table 2 summarizes the quantitative comparison with several state-of-the-art approaches, including CNN-based models, attention-enhanced networks, and recent multimodal fusion architectures. Across the three tasks, our model achieves favorable mean performance in Accuracy (ACC), Sensitivity (SEN), Specificity (SPEC), and Area Under the ROC Curve (AUC) under the present evaluation protocol, suggesting improved discriminative ability compared with the evaluated baselines.

For the AD vs. CN task, the proposed model reaches 94.22 ± 0.73% ACC and 97.74 ± 2.18% AUC, surpassing all baselines and highlighting its ability to reliably distinguish cognitively normal and AD subjects. In the AD vs. MCI task, which is inherently more challenging due to subtle cognitive changes in MCI, our method achieves 86.67 ± 4.70% ACC and 94.84 ± 0.94% AUC, outperforming all competing methods particularly in sensitivity (88.57 ± 9.92%), indicating improved detection of AD cases. For the CN vs. MCI task, our model maintains balanced performance with 75.67 ± 2.21% ACC and 82.71 ± 8.09% AUC, obtaining the best mean values among the evaluated methods under this setting.

Overall, these results suggest that the integration of multimodal information and the proposed network design may contribute to more discriminative representations under the current experimental setting, supporting improved classification under subtle inter-group differences. The observed improvements across the three tasks further indicate the potential value of the proposed approach for computer-aided assessment of AD-related neuroimaging patterns. The paired *t*-tests on fold-wise AUC values showed that CGMF-Net achieved statistically higher AUC than the best competing baseline in all three classification tasks (p<0.05).

### 3.2. Clinical Score Regression Performance

We evaluate the proposed model on three clinical score regression tasks—ADAS13, CDRSB, and MMSE—using the ADNI-2 cohort with five-fold cross-validation at the subject level. Table 3 presents the quantitative comparison with several baseline methods, including M3T, ResNet18, LBM, DM2L, MWAN, and CTA.

Across the three regression tasks, our model achieves competitive or favorable performance in both Pearson correlation coefficient (CC) and root mean squared error (RMSE) compared with the evaluated baselines. Specifically, for ADAS13, the proposed model reaches 0.86 ± 0.01 CC and 6.39 ± 0.37 RMSE, surpassing all baselines. For CDRSB, it achieves 0.86 ± 0.02 CC and 1.22 ± 0.11 RMSE, outperforming prior approaches, including MWAN (0.86 ± 0.05 CC, 1.25 ± 0.14 RMSE). For MMSE, the model obtains 0.83 ± 0.04 CC and 1.87 ± 0.10 RMSE, exceeding the best competing methods (MWAN/CTA 0.81 CC, 1.93–1.97 RMSE).

These results demonstrate that integrating multimodal MRI and PET information allows the model to capture continuous cognitive variations more accurately than single-task or single-modality baselines. The experiment confirms that the proposed approach provides both higher predictive accuracy and more stable regression performance across all three clinical scores, highlighting the effectiveness of our multimodal representation for clinical score regression.

### 3.3. Cross-Dataset Generalization

We further evaluate the generalization capability of the proposed model by training on ADNI-2 and testing on ADNI-1 for both classification and clinical score regression tasks. Table 4 and Table 5 summarize the cross-dataset performance of our method compared with multiple state-of-the-art approaches.

For classification tasks, our model consistently outperforms competing methods across all three tasks. Specifically, for AD vs. CN, the proposed model achieves 92.08 ± 2.43% ACC and 97.19 ± 0.51% AUC, surpassing all baselines, including HAMMF and CGANC. In the AD vs. MCI task, our approach attains 83.60 ± 4.08% ACC and 93.31 ± 1.91% AUC, demonstrating superior sensitivity and balanced performance compared to other methods. For CN vs. MCI, the model reaches 74.83 ± 4.62% ACC and 82.27 ± 4.30% AUC, outperforming the second-best baselines by clear margins.

For clinical score regression, the proposed method also demonstrates strong cross-dataset robustness. In ADAS13 prediction, the model achieves 0.86 ± 0.01 CC and 6.93 ± 0.32 RMSE, exceeding all baselines. For CDRSB, it attains 0.82 ± 0.01 CC and 1.53 ± 0.03 RMSE, and for MMSE, 0.80 ± 0.02 CC and 2.07 ± 0.11 RMSE, consistently outperforming the best competing methods, such as MWAN and CTA.

Overall, these results demonstrate that the proposed multimodal framework generalizes effectively to unseen cohorts, maintaining high predictive accuracy for both disease classification and clinical score regression. The model’s ability to transfer knowledge from ADNI-2 to ADNI-1 confirms the robustness and practical utility of the learned representations in cross-cohort applications.

### 3.4. Ablation Study

We conduct an ablation study to evaluate the contribution of different model components, feature-layer settings, auxiliary score supervision, and input modalities on three classification tasks (AD vs. CN, AD vs. MCI, CN vs. MCI) using the ADNI-2 cohort. Table 6 reports the quantitative results for module-level ablation, including variants without CMSGate, HSIC, and the regression head. Our full model consistently outperforms all ablated variants across all metrics. Removing the CMSGate module leads to notable drops in ACC and SPEC, highlighting its role in enhancing cross-modal feature integration. Similarly, omitting the HSIC branch reduces AUC and sensitivity, indicating its importance in reducing redundant coupling between bidirectional representations and improving discriminability. Excluding the regression head slightly decreases performance on AD vs. MCI and CN vs. MCI, confirming that auxiliary clinical supervision contributes to improved classification.

To further justify the selection of multi-scale feature layers, we conduct a feature-layer ablation study by comparing different combinations of Layer2, Layer3, and Layer4 features. As shown in Table 7, the proposed Layer3+4 setting achieves the best overall performance across all three classification tasks. Compared with using Layer4 alone, the joint Layer3+4 setting further improves ACC and AUC, indicating that intermediate semantic information from Layer3 provides complementary cues to the deeper Layer4 features. In contrast, using Layer2 or Layer3 alone leads to clear performance degradation, especially on the more challenging CN vs. MCI task, suggesting that single-scale or shallow representations are insufficient for stable discrimination between clinically adjacent categories. Although the Layer2+3+4 variant shows competitive results on the relatively easier AD vs. CN task, it does not outperform the Layer3+4 setting in any metric and exhibits clear degradation on the more challenging AD vs. MCI and CN vs. MCI tasks. This suggests that directly incorporating Layer2 may introduce low-level texture or redundant responses that are less consistently aligned across MRI and PET, thereby weakening stable cross-modal discrimination. Therefore, Layer3+4 was selected as a balanced design that preserves intermediate structural cues from Layer3 while retaining high-level task-related semantics from Layer4.

We also evaluate the influence of different auxiliary clinical scores on classification performance. As reported in Table 8, using MMSE as the auxiliary score supervision consistently achieves the best overall results across the three tasks. Compared with ADAS13 and CDRSB, MMSE provides more direct supervision related to global cognitive status and is more closely aligned with diagnostic discrimination in the current classification setting. ADAS13 and CDRSB still provide useful clinical guidance, but their performance is less stable, particularly on AD vs. MCI and CN vs. MCI, where the inter-class differences are more subtle. This may be because ADAS13 and CDRSB reflect different aspects of cognitive impairment and functional severity, which are not always optimally aligned with the binary classification boundaries. Therefore, MMSE is adopted as the classification-oriented auxiliary supervision signal in our final model.

**Figure 4:**
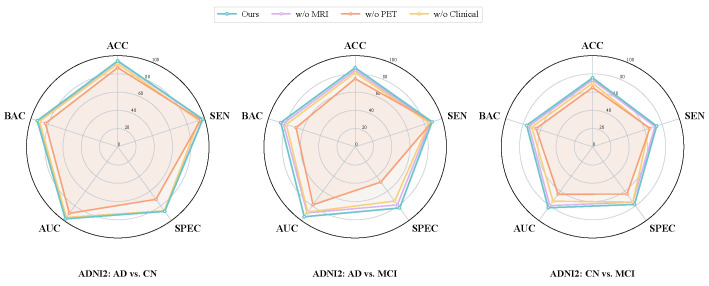
Radar plots of the modality ablation study on the ADNI-2 dataset. The figure compares the performance of the full model and its ablated variants across three binary classification tasks, including AD vs. CN, AD vs. MCI, and CN vs. MCI, in terms of ACC, SEN, SPEC, AUC, and BAC. Each radial axis ranges from 0 to 100%. Here, Ours denotes the full model, while w/o MRI, w/o PET, and w/o Clinical denote the variants without MRI, PET, and clinical score supervision, respectively.

We further investigate modality-level contributions using both quantitative results and radar plots (Table 9 and Figure 4). Excluding MRI, PET, or clinical information consistently reduces overall performance across all tasks, with the largest drops observed when PET data is removed, particularly in AD vs. MCI. This analysis confirms that each modality provides complementary information, and the full combination of MRI, PET, and clinical features is essential to achieve optimal classification performance. Collectively, the ablation experiments demonstrate that the proposed modules, the Layer3+4 multi-scale design, MMSE-based auxiliary supervision, and multimodal inputs are all critical for the robust and discriminative performance of the model. The w/o Clinical setting removes the auxiliary clinical-score supervision and can also be regarded as a sensitivity analysis without cognitive-score supervision. Although removing this auxiliary supervision decreases performance compared with the full model, CGMF-Net still maintains competitive classification results using paired sMRI and FDG-PET inputs. This suggests that the diagnostic prediction is not solely driven by label-proximal cognitive-score supervision, while MMSE-based auxiliary supervision can contribute to more discriminative shared representation learning.

### 3.5. Parameter Sensitivity and Visualization

**Figure 5:**
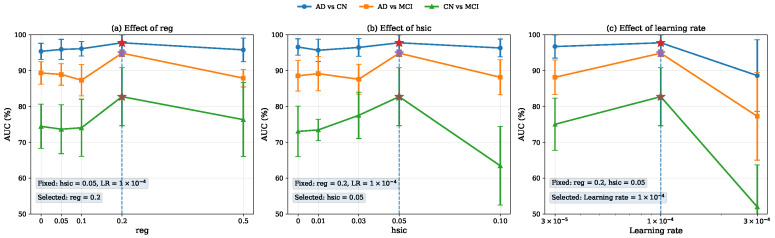
Parameter sensitivity analysis of the proposed model in terms of AUC (%). (**a**) Effect of the regression loss weight reg with hsic=0.05 and $\mathrm{LR}=10^{-4}$. (**b**) Effect of the HSIC regularization weight hsic with reg=0.2 and $\mathrm{LR}=10^{-4}$. (**c**) Effect of the learning rate LR with reg=0.2 and hsic=0.05. The dashed vertical line indicates the selected parameter setting in each sweep. Stars mark the maximum AUC value for each curve.

We analyze the sensitivity of the proposed model to key hyperparameters and visualize its interpretability using Grad-CAM. Figure 5 presents the effect of regression loss weight reg, HSIC regularization weight hsic, and learning rate LR on the AUC of three classification tasks (AD vs. CN, AD vs. MCI, CN vs. MCI) on the ADNI-2 cohort. The results show that moderate values of reg=0.2 and hsic=0.05, along with a learning rate of $\mathrm{LR}=10^{-4}$, yield the best trade-off between convergence stability and predictive performance. AUC decreases slightly when deviating from these settings, indicating that the model is robust to small hyperparameter variations but benefits from appropriately balanced auxiliary regression and HSIC supervision.

**Figure 6:**
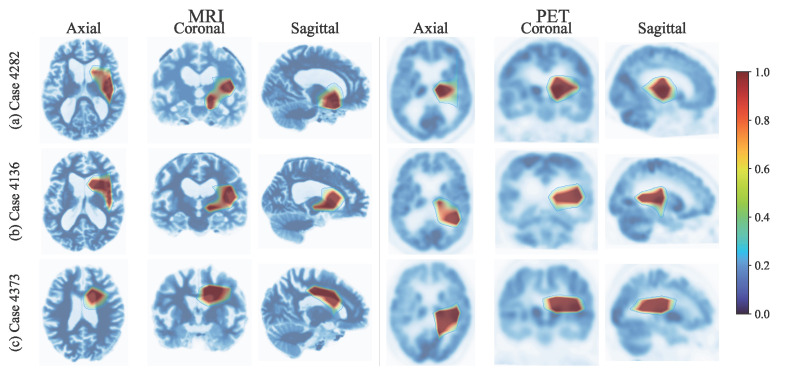
Grad-CAM visualizations of three representative cases across MRI and PET modalities. For each case, axial, coronal, and sagittal views are shown to illustrate the spatial regions contributing to the model’s prediction. These Grad-CAM maps were used as qualitative post hoc visualizations to inspect modality-specific activation patterns. The highlighted regions appear broadly consistent with AD-related medial temporal and temporoparietal areas, but they should not be interpreted as atlas-based quantitative localization of Alzheimer’s-related structures. For visualization, the Grad-CAM maps were normalized, smoothed with a Gaussian filter (σ=0.8), restricted to the MRI-derived brain mask to exclude non-brain areas, and displayed using a threshold of 0.22 before being overlaid on the corresponding MRI or PET slices.

To further interpret the learned multimodal representations, we generate Grad-CAM visualizations for three representative cases using MRI and PET inputs (Figure 6). For each case, axial, coronal, and sagittal views are shown to illustrate the spatial regions contributing to the model’s prediction. The MRI and PET activation patterns suggest complementary information, with PET responses tending to emphasize metabolic abnormalities and MRI responses tending to highlight structural variations. The highlighted regions appear broadly located in AD-related medial temporal and temporoparietal areas; however, these Grad-CAM maps are qualitative post hoc visualizations and should not be interpreted as atlas-based quantitative localization or direct evidence of causal biomarkers.

Overall, these analyses validate the selected hyperparameter settings and demonstrate that the model not only achieves robust predictive performance but also provides interpretable spatial insights into modality-specific contributions.

## 4. Discussion

This study proposes CGMF-Net, a cross-modal gated multi-task learning framework for integrating structural and metabolic neuroimaging biomarkers in Alzheimer’s disease analysis. By jointly modeling paired sMRI and FDG-PET data, the proposed model aims to improve both AD classification and cognitive score estimation. The experimental results demonstrate that CGMF-Net achieves consistently strong performance across three classification tasks, namely AD vs. CN, AD vs. MCI, and CN vs. MCI, and also obtains competitive regression results for ADAS13, CDRSB, and MMSE. These findings suggest that structural atrophy and metabolic dysfunction provide complementary disease-related information and that their joint modeling can produce more informative representations than single-stream or conventional fusion-based approaches. Although some numerical improvements over competing methods are modest, these gains should be interpreted as incremental improvements in auxiliary computer-aided assessment under a unified paired-imaging protocol, and their practical clinical relevance still requires further validation in larger independent cohorts.

One important observation is that the proposed cross-modal gated learning strategy improves the quality of multimodal feature fusion. Existing multimodal AD methods often rely on direct concatenation, global fusion, or late-stage aggregation, which may introduce inconsistent or redundant responses from different modalities into the final representation. In contrast, CMSGate enhances cross-modal consistency before explicit fusion by strengthening responses that are supported by the complementary modality. The ablation results show that removing CMSGate leads to clear performance degradation, indicating that cross-modal consistency enhancement is beneficial for learning discriminative structural–metabolic representations. This result is consistent with the motivation that sMRI and FDG-PET should not simply be combined at the representation level but should be adaptively aligned and enhanced according to their complementary disease-related patterns.

The bi-directional cross-attention mechanism further contributes to the effectiveness of the proposed framework. Instead of modeling multimodal interaction in only one direction, CGMF-Net explicitly captures both MRI-to-PET and PET-to-MRI dependencies. This design allows the model to learn structure-guided metabolic information and metabolism-guided structural information simultaneously. In AD analysis, such bidirectional interaction is meaningful because structural degeneration and metabolic abnormality are closely related but not identical manifestations of disease progression. By preserving both interaction directions before constructing the final shared representation, the model can better capture complementary multimodal patterns associated with diagnostic status and cognitive impairment.

The multi-task learning design also plays an important role in improving representation quality. In the proposed framework, clinical score regression is used as an auxiliary task to guide the shared multimodal representation toward disease-severity-related variations. The results show that incorporating the regression branch improves classification performance compared with the variant without regression supervision. This indicates that continuous cognitive measures provide complementary information beyond categorical diagnostic labels. In particular, MMSE reflects global cognitive status and can help the model encode fine-grained clinical variations that may not be fully captured by class labels alone. Meanwhile, the separate regression evaluation on ADAS13, CDRSB, and MMSE further confirms that the learned representation is not only discriminative for classification but also clinically relevant for cognitive score estimation.

The HSIC regularization term provides an additional constraint for controlling redundant coupling between the two directional cross-modal representations. The ablation results indicate that removing HSIC reduces the overall performance, suggesting that explicitly regularizing the dependence between bidirectional representations helps preserve complementary information. This is particularly useful in multimodal fusion, where different interaction branches may otherwise learn highly overlapping information. By reducing unnecessary redundancy, HSIC encourages the model to maintain more diverse and informative directional representations, thereby improving both robustness and generalization.

The cross-dataset experiments further demonstrate the generalization ability of the proposed framework. When trained on ADNI-2 and tested on ADNI-1, CGMF-Net maintains favorable classification and regression performance compared with competing methods. This result is important because cohort-level distribution differences are common in neuroimaging studies, especially when data are collected under different acquisition protocols or population characteristics. The improved cross-cohort performance suggests that the proposed combination of cross-modal consistency enhancement, bidirectional interaction, multi-task supervision, and redundancy regularization can help learn more transferable multimodal representations.

The parameter sensitivity analysis shows that moderate values of the regression loss weight and HSIC regularization weight lead to better performance, while overly weak or overly strong auxiliary constraints may reduce the benefit of multi-task supervision or regularization. This indicates that the proposed model benefits from a balanced optimization strategy, where classification remains the primary objective while clinical score regression and HSIC regularization provide complementary guidance. In addition, the Grad-CAM visualizations show that MRI and PET contribute different but complementary activation patterns. MRI tends to highlight structural variations, whereas PET emphasizes metabolic abnormalities, supporting the interpretability of the proposed multimodal fusion strategy.

Despite these encouraging results, several limitations remain. First, this study was conducted using ADNI data, and although cross-dataset evaluation between ADNI-2 and ADNI-1 was performed, further validation on larger independent multi-center cohorts is still needed to assess the robustness of the model under broader clinical conditions. Second, the current framework focuses on subjects with paired sMRI and FDG-PET data. In real clinical scenarios, FDG-PET may be unavailable for some patients due to cost, accessibility, or radiation exposure, and therefore extending the framework to incomplete-modality or missing-modality settings would be valuable. Accordingly, the intended use of CGMF-Net should be considered as research-oriented or an auxiliary assessment in memory clinic settings where paired sMRI and FDG-PET are already available, rather than routine population screening; the w/o PET ablation provides an initial MRI-only reference, while explicit missing-modality inference remains future work. Third, although the model considers cognitive score estimation, the current implementation mainly uses baseline imaging data and does not explicitly model longitudinal disease trajectories. Incorporating longitudinal neuroimaging and clinical follow-up information may further improve the characterization of disease progression. Finally, the interpretability analysis is based mainly on qualitative post hoc Grad-CAM visualization rather than atlas-based region-level quantification; therefore, more quantitative neurobiological validation may be required to better connect model activation patterns with established AD-related brain regions and clinical findings.

In summary, the proposed CGMF-Net provides an effective framework for integrating structural and metabolic neuroimaging biomarkers for AD diagnosis and cognitive score estimation. By combining cross-modal consistency gating, bidirectional cross-attention, multi-task supervision, and HSIC regularization, the model learns discriminative and clinically relevant multimodal representations. The results indicate that this strategy has potential value for computer-aided diagnostic assessment and cognitive-status-related prediction in Alzheimer’s disease. Future work will focus on external multi-center validation, incomplete-modality learning, longitudinal modeling, and more clinically interpretable multimodal representation analysis.

## 5. Conclusions

In this study, we propose CGMF-Net, a cross-modal gated multi-task fusion framework for integrating paired sMRI and FDG-PET data in Alzheimer’s disease analysis. By combining multi-scale feature extraction, cross-modal similarity gating, bi-directional cross-attention, cognitive score supervision, and HSIC regularization, the proposed model enhances structural–metabolic consistency and learns discriminative multimodal representations for both AD classification and cognitive score estimation. Experiments on the ADNI cohort demonstrate that CGMF-Net achieves strong diagnostic performance, competitive cognitive score estimation, favorable cross-dataset generalization from ADNI-2 to ADNI-1, and consistent improvements in ablation and sensitivity analyses. These results suggest that cross-modal gated learning provides a useful strategy for integrating structural and metabolic neuroimaging biomarkers and may support computer-aided diagnostic assessment and cognitive-status-related prediction in AD. Future work will focus on external multi-center validation, incomplete-modality learning, longitudinal disease modeling, and more clinically interpretable multimodal representation analysis. 

## Figures and Tables

**Table 1:** Dataset statistics of ADNI-1 and ADNI-2 used in this study.

Dataset		AD	MCI	CN	Total
ADNI-1	F/M	36/48	62/121	38/55	
	Age	75.84 ± 7.38	74.71 ± 7.21	75.76 ± 4.86	
	Count	84	183	93	360
ADNI-2	F/M	58/78	146/182	131/114	
	Age	74.49 ± 8.20	71.69 ± 7.34	72.82 ± 6.04	
	Count	136	328	245	709
**Total**		**220**	**511**	**338**	**1069**

**Table 2:** Performance comparison of different methods on three classification tasks, including AD vs. CN, AD vs. MCI, and CN vs. MCI. The best result in each column is highlighted in bold, and the second-best result is underlined. All values are reported as mean ± standard deviation (%).

Method	AD vs. CN				AD vs. MCI				CN vs. MCI			
	ACC	SEN	SPEC	AUC	ACC	SEN	SPEC	AUC	ACC	SEN	SPEC	AUC
ResNet18 [20]	90.69 ± 5.92	96.92 ± 1.48	76.19 ± 16.15	95.66 ± 3.31	79.33 ± 7.69	78.00 ± 18.06	82.00 ± 15.25	90.40 ± 2.43	64.62 ± 6.38	59.05 ± 18.32	71.11 ± 16.85	73.44 ± 3.43
MMSDL [24]	90.69 ± 7.48	94.05 ± 6.73	84.76 ± 10.86	95.47 ± 3.67	81.00 ± 5.96	81.00 ± 12.32	81.00 ± 19.49	90.86 ± 6.09	63.08 ± 8.23	62.86 ± 12.33	63.33 ± 26.82	67.67 ± 11.36
D-MAFF [25]	92.41 ± 2.61	96.22 ± 4.10	85.71 ± 8.25	97.73 ± 2.48	83.33 ± 2.04	85.00 ± 3.06	80.00 ± 7.91	88.20 ± 2.86	72.31 ± 4.21	69.52 ± 10.96	75.56 ± 19.08	80.11 ± 5.26
MENet [26]	91.38 ± 3.45	96.76 ± 2.26	81.90 ± 7.06	93.49 ± 4.37	80.67 ± 4.94	81.00 ± 8.40	80.00 ± 9.35	85.55 ± 3.39	66.67 ± 6.28	67.62 ± 16.97	65.56 ± 30.78	75.03 ± 5.28
HAMMF [13]	93.10 ± 4.40	96.76 ± 2.26	86.67 ± 10.32	95.57 ± 4.07	81.33 ± 5.70	83.50 ± 13.06	77.00 ± 11.51	89.80 ± 1.69	70.77 ± 9.70	68.57 ± 12.87	73.33 ± 9.94	76.72 ± 8.35
MACFNet [12]	91.38 ± 3.23	95.68 ± 5.60	83.81 ± 10.96	94.93 ± 3.53	82.67 ± 1.90	85.00 ± 9.84	78.00 ± 14.83	90.11 ± 2.73	72.31 ± 2.81	70.48 ± 10.32	74.44 ± 11.52	78.31 ± 4.54
MDL-Net [5]	92.93 ± 2.01	96.34 ± 3.02	86.80 ± 6.06	96.03 ± 3.40	82.47 ± 3.59	87.46 ± 2.11	72.86 ± 11.63	87.83 ± 2.79	74.12 ± 9.94	72.07 ± 12.56	74.17 ± 27.86	80.12 ± 8.00
IMDL [27]	91.88 ± 3.00	96.33 ± 4.43	83.76 ± 7.76	96.02 ± 2.78	80.46 ± 3.21	84.82 ± 7.05	72.12 ± 13.81	89.19 ± 2.19	63.14 ± 4.47	60.00 ± 18.22	66.67 ± 29.76	71.08 ± 8.47
DHFWLSL [10]	92.67 ± 2.01	97.56 ± 1.71	83.78 ± 5.71	95.68 ± 1.43	80.70 ± 3.37	84.83 ± 7.64	72.83 ± 13.39	88.95 ± 3.28	62.35 ± 8.25	64.44 ± 22.31	60.00 ± 35.67	70.93 ± 9.41
CGANC [9]	93.20 ± 2.52	96.76 ± 3.07	86.83 ± 5.92	96.46 ± 1.57	81.70 ± 4.48	86.34 ± 6.97	72.67 ± 12.02	89.77 ± 3.54	65.10 ± 5.78	56.30 ± 21.34	75.00 ± 25.17	72.75 ± 6.12
Ours	**94.22 ± 0.73**	**97.96 ± 2.89**	**87.51 ± 6.69**	**97.74 ± 2.18**	**86.67 ± 4.70**	**88.57 ± 9.92**	**82.93 ± 12.09**	**94.84 ± 0.94**	**75.67 ± 2.21**	**73.78 ± 16.08**	**78.11 ± 7.66**	**82.71 ± 8.09**

**Table 3:** Performance comparison of different methods on three clinical score regression tasks, including ADAS13, CDRSB, and MMSE. The best result in each column is highlighted in bold, and the second-best result is underlined. ↑ denotes higher values indicate better performance for CC (correlation coefficient), while ↓ denotes lower values indicate better performance for RMSE (root mean square error).

Method	ADAS13		CDRSB		MMSE	
	CC↑	RMSE↓	CC↑	RMSE↓	CC↑	RMSE↓
M3T [4]	0.73 ± 0.12	8.85 ± 0.91	0.71 ± 0.05	1.72 ± 0.23	0.68 ± 0.09	2.49 ± 0.11
ResNet18 [20]	0.83 ± 0.05	6.93 ± 0.82	0.83 ± 0.06	1.32 ± 0.16	0.79 ± 0.06	2.03 ± 0.25
LBM [28]	0.85 ± 0.02	6.46 ± 0.52	0.84 ± 0.02	1.30 ± 0.12	0.79 ± 0.03	2.02 ± 0.19
DM2L [15]	0.81 ± 0.07	7.26 ± 1.17	0.80 ± 0.08	1.41 ± 0.32	0.76 ± 0.08	2.09 ± 0.33
MWAN [16]	0.84 ± 0.06	6.79 ± 0.70	0.86 ± 0.05	1.25 ± 0.14	0.81 ± 0.04	1.93 ± 0.12
CTA [18]	0.85 ± 0.04	6.62 ± 0.36	0.84 ± 0.05	1.28 ± 0.19	0.81 ± 0.04	1.97 ± 0.14
Ours	**0.86 ± 0.01**	**6.39 ± 0.37**	**0.86 ± 0.02**	**1.22 ± 0.11**	**0.83 ± 0.04**	**1.87 ± 0.10**

**Table 4:** Cross-dataset generalization performance comparison of different methods on three classification tasks, including AD vs. CN, AD vs. MCI, and CN vs. MCI, where the models are trained on ADNI-2 and tested on ADNI-1. The best result in each column is highlighted in bold, and the second-best result is underlined. All values are reported as mean ± standard deviation (%).

Method	AD vs. CN				AD vs. MCI				CN vs. MCI			
	ACC	SEN	SPEC	AUC	ACC	SEN	SPEC	AUC	ACC	SEN	SPEC	AUC
ResNet18 [20]	85.91 ± 2.93	90.87 ± 3.19	80.48 ± 8.70	93.31 ± 0.95	73.81 ± 3.19	69.52 ± 8.44	78.10 ± 8.96	83.76 ± 3.84	65.83 ± 4.63	77.55 ± 11.40	53.62 ± 20.29	72.90 ± 3.96
MMSDL [24]	88.64 ± 2.78	89.13 ± 8.13	88.10 ± 5.63	95.41 ± 1.17	76.43 ± 3.14	79.05 ± 13.33	73.81 ± 16.29	87.12 ± 2.39	58.75 ± 5.21	77.55 ± 12.65	39.15 ± 21.13	65.58 ± 2.82
D-MAFF [25]	88.86 ± 1.67	89.57 ± 1.63	88.10 ± 2.61	94.45 ± 1.41	80.48 ± 2.33	81.43 ± 5.91	79.52 ± 3.87	87.16 ± 3.16	70.42 ± 2.76	79.18 ± 7.89	61.28 ± 12.14	77.02 ± 3.27
MENet [26]	86.36 ± 4.25	83.48 ± 7.35	89.52 ± 2.43	95.07 ± 2.19	75.95 ± 4.79	84.76 ± 8.86	67.14 ± 16.66	85.06 ± 2.93	60.42 ± 2.28	84.49 ± 10.85	35.32 ± 13.50	69.60 ± 3.16
HAMMF [13]	89.55 ± 2.82	88.26 ± 2.61	90.95 ± 3.50	95.59 ± 2.18	79.29 ± 2.45	78.57 ± 6.90	80.00 ± 9.71	85.94 ± 2.99	70.62 ± 2.75	81.22 ± 5.83	59.57 ± 11.10	81.42 ± 3.61
MACFNet [12]	86.14 ± 3.40	81.74 ± 5.77	90.95 ± 2.78	94.43 ± 1.14	77.62 ± 3.64	79.05 ± 4.10	76.19 ± 9.28	84.88 ± 1.75	68.33 ± 4.87	78.37 ± 3.79	57.87 ± 7.78	77.52 ± 4.92
MDL-Net [5]	87.73 ± 1.82	86.96 ± 4.35	88.57 ± 5.71	95.21 ± 1.30	78.33 ± 3.94	79.05 ± 3.16	77.62 ± 6.83	85.67 ± 3.45	68.33 ± 4.82	79.59 ± 15.27	56.60 ± 25.59	78.28 ± 4.44
IMDL [27]	88.30 ± 3.85	90.71 ± 7.35	85.60 ± 5.43	94.11 ± 3.38	76.43 ± 4.60	80.10 ± 2.13	64.76 ± 9.09	85.63 ± 1.87	65.42 ± 4.72	86.94 ± 11.87	42.98 ± 19.52	74.66 ± 2.35
DHFWLSL [10]	87.27 ± 3.17	86.96 ± 3.64	87.62 ± 5.91	94.50 ± 1.04	75.24 ± 3.05	84.76 ± 6.83	65.71 ± 12.65	83.84 ± 1.61	63.33 ± 8.34	85.31 ± 9.78	40.43 ± 24.74	68.72 ± 9.07
CGANC [9]	89.09 ± 2.23	87.39 ± 4.84	90.95 ± 2.78	94.53 ± 0.32	76.43 ± 2.65	80.00 ± 8.86	72.86 ± 8.60	84.42 ± 3.81	69.38 ± 2.24	76.39 ± 12.24	62.36 ± 15.14	73.83 ± 1.93
Ours	**92.08 ± 2.43**	**92.50 ± 6.12**	**91.67 ± 2.64**	**97.19 ± 0.51**	**83.60 ± 4.08**	**84.80 ± 8.91**	**82.40 ± 5.99**	**93.31 ± 1.91**	**74.83 ± 4.62**	**87.66 ± 10.90**	**63.00 ± 11.49**	**82.27 ± 4.30**

**Table 5:** Generalization performance of different methods when trained on ADNI-2 and tested on ADNI-1 for three clinical score regression tasks, including ADAS13, CDRSB, and MMSE. The best result in each column is highlighted in bold, and the second-best result is underlined. ↑ denotes higher values indicate better performance for CC (correlation coefficient), while ↓ denotes lower values indicate better performance for RMSE (root mean square error).

Method	ADAS13		CDRSB		MMSE	
	CC↑	RMSE↓	CC↑	RMSE↓	CC↑	RMSE↓
M3T [4]	0.78 ± 0.02	8.09 ± 0.43	0.70 ± 0.02	2.11 ± 0.08	0.68 ± 0.01	2.46 ± 0.05
ResNet18 [20]	0.77 ± 0.02	7.60 ± 0.25	0.73 ± 0.02	1.92 ± 0.07	0.73 ± 0.02	2.48 ± 0.09
LBM [28]	0.75 ± 0.03	8.07 ± 0.52	0.70 ± 0.02	2.02 ± 0.07	0.73 ± 0.02	2.53 ± 0.13
DM2L [15]	0.76 ± 0.06	8.13 ± 1.03	0.72 ± 0.06	1.82 ± 0.23	0.72 ± 0.07	2.44 ± 0.29
MWAN [16]	0.77 ± 0.03	7.31 ± 0.45	0.78 ± 0.05	1.59 ± 0.17	0.79 ± 0.04	2.30 ± 0.15
CTA [18]	0.79 ± 0.02	7.25 ± 0.33	0.77 ± 0.02	1.81 ± 0.09	0.76 ± 0.06	2.38 ± 0.23
Ours	**0.86 ± 0.01**	**6.93 ± 0.32**	**0.82 ± 0.01**	**1.53 ± 0.03**	**0.80 ± 0.02**	**2.07 ± 0.11**

**Table 6:** Results of the module ablation study on three classification tasks, including AD vs. CN, AD vs. MCI, and CN vs. MCI. The best result in each column is highlighted in bold, and the second-best result is underlined. All values are reported as mean ± standard deviation (%). Here, “w/o” denotes the variant without the corresponding module.

Ablation Variant	AD vs. CN				AD vs. MCI				CN vs. MCI			
	ACC	SEN	SPEC	AUC	ACC	SEN	SPEC	AUC	ACC	SEN	SPEC	AUC
Ours	**94.22 ± 0.73**	**97.96 ± 2.89**	**87.51 ± 6.69**	**97.74 ± 2.18**	**86.67 ± 4.70**	**88.57 ± 9.92**	**82.93 ± 12.09**	**94.84 ± 0.94**	**75.67 ± 2.21**	**73.78 ± 16.08**	**78.11 ± 7.66**	**82.71 ± 8.09**
w/o CMSGate	91.09 ± 3.81	96.33 ± 3.03	81.75 ± 13.92	95.99 ± 2.07	81.21 ± 6.22	85.55 ± 13.79	72.75 ± 25.52	88.82 ± 4.08	64.31 ± 7.39	62.96 ± 17.95	65.83 ± 22.71	71.54 ± 9.09
w/o HSIC	91.08 ± 2.96	97.14 ± 5.32	80.24 ± 11.85	95.89 ± 2.85	82.95 ± 6.02	85.21 ± 8.94	78.65 ± 17.28	89.62 ± 4.53	68.24 ± 8.48	63.70 ± 13.71	73.33 ± 20.54	75.37 ± 11.23
w/o Regression	90.83 ± 2.12	93.49 ± 4.43	86.01 ± 10.95	96.06 ± 2.77	84.21 ± 3.41	88.30 ± 8.09	74.18 ± 11.55	89.42 ± 3.52	63.14 ± 7.12	61.48 ± 24.65	65.00 ± 34.18	75.03 ± 7.93

**Table 7:** Results of the feature-layer ablation study on three classification tasks, including AD vs. CN, AD vs. MCI, and CN vs. MCI. The best result in each column is highlighted in bold, and the second-best result is underlined. All values are reported as mean ± standard deviation (%). Here, “✔” indicates that the corresponding feature layer is used.

Variant	Feature Layer			AD vs. CN				AD vs. MCI				CN vs. MCI			
	Layer2	Layer3	Layer4	ACC	SEN	SPEC	AUC	ACC	SEN	SPEC	AUC	ACC	SEN	SPEC	AUC
Layer3+4 (Ours)		✔	✔	**94.22 ± 0.73**	**97.96 ± 2.89**	**87.51 ± 6.69**	**97.74 ± 2.18**	**86.67 ± 4.70**	**88.57 ± 9.92**	**82.93 ± 12.09**	**94.84 ± 0.94**	**75.67 ± 2.21**	**73.78 ± 16.08**	**78.11 ± 7.66**	**82.71 ± 8.09**
Layer4			✔	91.07 ± 5.38	93.47 ± 10.24	86.77 ± 4.18	96.59 ± 2.73	80.22 ± 6.30	88.47 ± 8.94	60.19 ± 16.22	89.06 ± 3.15	71.37 ± 4.07	71.85 ± 13.25	70.83 ± 11.02	80.37 ± 4.25
Layer3		✔		85.58 ± 5.56	93.47 ± 7.27	71.32 ± 11.53	92.55 ± 4.75	77.19 ± 4.83	85.99 ± 14.37	60.26 ± 24.53	84.85 ± 6.65	58.82 ± 8.43	65.19 ± 25.74	51.67 ± 40.57	65.43 ± 5.45
Layer2	✔			73.74 ± 7.89	71.84 ± 14.02	77.17 ± 13.77	82.90 ± 3.90	71.69 ± 4.50	81.61 ± 7.73	43.02 ± 20.59	76.89 ± 4.53	54.51 ± 4.68	69.63 ± 41.28	37.50 ± 49.56	63.50 ± 7.34
Layer2+3	✔	✔		86.87 ± 6.04	91.84 ± 9.89	77.96 ± 8.19	93.21 ± 4.26	74.45 ± 4.99	83.32 ± 14.29	57.41 ± 31.04	85.76 ± 5.29	58.04 ± 5.30	71.85 ± 24.37	42.50 ± 25.92	62.41 ± 3.80
Layer2+3+4	✔	✔	✔	92.91 ± 2.73	95.10 ± 4.23	86.97 ± 3.71	96.14 ± 2.22	80.21 ± 4.91	87.05 ± 12.28	66.77 ± 21.71	88.58 ± 4.36	61.96 ± 8.94	60.00 ± 28.38	64.17 ± 30.98	66.91 ± 8.42

**Table 8:** Results of the auxiliary score supervision ablation study on three classification tasks, including AD vs. CN, AD vs. MCI, and CN vs. MCI. The best result in each column is highlighted in bold, and the second-best result is underlined. All values are reported as mean ± standard deviation (%).

Auxiliary Score	AD vs. CN				AD vs. MCI				CN vs. MCI			
	ACC	SEN	SPEC	AUC	ACC	SEN	SPEC	AUC	ACC	SEN	SPEC	AUC
MMSE (Ours)	**94.22 ± 0.73**	**97.96 ± 2.89**	**87.51 ± 6.69**	**97.74 ± 2.18**	**86.67 ± 4.70**	**88.57 ± 9.92**	**82.93 ± 12.09**	**94.84 ± 0.94**	**75.67 ± 2.21**	**73.78 ± 16.08**	**78.11 ± 7.66**	**82.71 ± 8.09**
ADAS13	89.95 ± 4.60	94.69 ± 1.83	81.31 ± 13.99	94.11 ± 4.97	80.25 ± 3.05	88.13 ± 9.19	64.59 ± 15.66	88.45 ± 3.39	66.27 ± 6.71	71.11 ± 9.59	60.83 ± 23.12	74.91 ± 5.44
CDRSB	90.01 ± 5.16	91.84 ± 12.58	86.77 ± 9.99	96.67 ± 2.42	80.69 ± 4.07	82.51 ± 4.94	77.20 ± 12.95	87.80 ± 5.38	62.75 ± 7.84	54.81 ± 28.13	71.67 ± 35.77	72.01 ± 5.36

**Table 9:** Results of the modality ablation study on three classification tasks. All values are reported as percentages (%). Bold and underlined values indicate the best and second-best results, respectively.

Modality Ablation	AD vs. CN					AD vs. MCI					CN vs. MCI				
	ACC	SEN	SPEC	AUC	BAC	ACC	SEN	SPEC	AUC	BAC	ACC	SEN	SPEC	AUC	BAC
Ours	**94.22**	**97.96**	**87.51**	**97.74**	**92.74**	**86.67**	**88.57**	**82.93**	**94.84**	**85.75**	**75.67**	**73.78**	**78.11**	**82.71**	**75.95**
w/o MRI	93.70	97.55	86.75	95.77	92.15	83.95	86.71	78.76	89.59	82.73	73.33	71.85	75.00	79.81	73.43
w/o PET	86.87	95.51	71.19	90.18	83.35	74.68	88.55	47.78	78.50	68.16	65.10	65.93	64.17	64.29	65.05
w/o Clinical	91.10	93.52	86.80	95.90	90.16	81.21	85.20	73.57	88.52	79.39	69.80	65.19	75.00	73.77	70.09

## Data Availability

The data used in this study are available from the Alzheimer’s Disease Neuroimaging Initiative (ADNI) database upon application and approval. Access to ADNI data can be requested through the official ADNI data access platform and is subject to the ADNI data use agreement.

## Abbreviations

The following abbreviations are used in this manuscript:

AD	Alzheimer’s disease
MCI	Mild cognitive impairment
CN	Cognitively normal control
sMRI	Structural magnetic resonance imaging
FDG-PET	Fluorodeoxyglucose positron emission tomography
ADNI	Alzheimer’s Disease Neuroimaging Initiative
CMSGate	Cross-Modal Similarity Gate
HSIC	Hilbert–Schmidt independence criterion
MMSE	Mini-Mental State Examination
ADAS13	Alzheimer’s Disease Assessment Scale–Cognitive Subscale
CDRSB	Clinical Dementia Rating Sum of Boxes
ACC	Accuracy
SEN	Sensitivity
SPEC	Specificity
AUC	Area under the receiver operating characteristic curve
RMSE	Root mean squared error
CC	Pearson correlation coefficient

## References

[1] Alzheimer’s Association (2024). 2024 Alzheimer’s disease facts and figures. Alzheimer’S Dement..

[2] van Oostveen W.M., de Lange E.C.M. (2021). Imaging Techniques in Alzheimer’s Disease: A Review of Applications in Early Diagnosis and Longitudinal Monitoring. Int. J. Mol. Sci..

[3] Sharma R., Goel T., Tanveer M., Lin C.T., Murugan R. (2023). Deep-Learning-Based Diagnosis and Prognosis of Alzheimer’s Disease: A Comprehensive Review. IEEE Trans. Cogn. Dev. Syst..

[4] Zhang D., Shen D. (2012). Multi-modal Multi-task Learning for Joint Prediction of Multiple Regression and Classification Variables in Alzheimer’s Disease. NeuroImage.

[5] Qiu Z., Yang P., Xiao C., Wang S., Xiao X., Qin J., Liu C.M., Wang T., Lei B. (2024). 3D Multimodal Fusion Network with Disease-Induced Joint Learning for Early Alzheimer’s Disease Diagnosis. IEEE Trans. Med. Imaging.

[6] Guan Y., Wang W., Chen J., Yang P., Xu J., Qi J. (2026). A Survey of Multimodal Fusion for Alzheimer’s Disease Prediction: A New Taxonomy and Trends. Inf. Fusion.

[7] Li Y., Daho M.E.H., Conze P.H., Zeghlache R., Le Boité H., Tadayoni R., Cochener B., Lamard M., Quellec G. (2024). A review of deep learning-based information fusion techniques for multimodal medical image classification. Comput. Biol. Med..

[8] Duan J., Xiong J., Li Y., Ding W. (2024). Deep learning based multimodal biomedical data fusion: An overview and comparative review. Inf. Fusion.

[9] Choudhury C., Goel T., Tanveer M. (2024). A Coupled-GAN Architecture to Fuse MRI and PET Image Features for Multi-stage Classification of Alzheimer’s Disease. Inf. Fusion.

[10] Luo Y., Chen H., Yin T., Horng S.J., Li T. (2024). Dual Hypergraphs with Feature Weighted and Latent Space Learning for the Diagnosis of Alzheimer’s Disease. Inf. Fusion.

[11] Kong Z., Zhang M., Zhu W., Yi Y., Wang T., Zhang B. (2022). Multi-modal data Alzheimer’s disease detection based on 3D convolution. Biomed. Signal Process. Control.

[12] Tang C., Xi M., Sun J., Wang S., Zhang Y., Initiative A.D.N. (2024). MACFNet: Detection of Alzheimer’s Disease via Multiscale Attention and Cross-Enhancement Fusion Network. Comput. Methods Programs Biomed..

[13] Liu X., Li W., Miao S., Liu F., Han K., Bezabih T.T. (2024). HAMMF: Hierarchical Attention-based Multi-task and Multi-modal Fusion Model for Computer-aided Diagnosis of Alzheimer’s Disease. Comput. Biol. Med..

[14] Caruana R. (1997). Multitask Learning. Mach. Learn..

[15] Liu M., Zhang J., Adeli E., Shen D. (2019). Joint Classification and Regression via Deep Multi-Task Multi-Channel Learning for Alzheimer’s Disease Diagnosis. IEEE Trans. Biomed. Eng..

[16] Lian C., Liu M., Wang L., Shen D. (2022). Multi-Task Weakly-Supervised Attention Network for Dementia Status Estimation with Structural MRI. IEEE Trans. Neural Netw. Learn. Syst..

[17] El-Sappagh S., Abuhmed T., Islam S.M.R., Kwak K.S. (2020). Multimodal Multitask Deep Learning Model for Alzheimer’s Disease Progression Detection Based on Time Series Data. Neurocomputing.

[18] Li S., Zhang Y., Zou C., Zhang L., Li F., Liu Q. (2025). Transformer Attention-Based Neural Network for Cognitive Score Estimation from sMRI Data. Comput. Biol. Med..

[19] Lei B., Liang E., Yang M., Yang P., Zhou F., Tan E.L., Lei Y., Liu C.M., Wang T., Xiao X. (2022). Predicting clinical scores for Alzheimer’s disease based on joint and deep learning. Expert Syst. Appl..

[20] Hara K., Kataoka H., Satoh Y. Can Spatiotemporal 3D CNNs Retrace the History of 2D CNNs and ImageNet?. Proceedings of the IEEE/CVF Conference on Computer Vision and Pattern Recognition.

[21] Vaswani A., Shazeer N., Parmar N., Uszkoreit J., Jones L., Gomez A.N., Kaiser L., Polosukhin I. Attention Is All You Need. Proceedings of the Advances in Neural Information Processing Systems.

[22] Huber P.J. (1964). Robust Estimation of a Location Parameter. Ann. Math. Stat..

[23] Gretton A., Bousquet O., Smola A., Schölkopf B. (2005). Measuring Statistical Dependence with Hilbert-Schmidt Norms. Proceedings of the International Conference on Algorithmic Learning Theory.

[24] Abdelaziz M., Wang T., Anwaar W., Elazab A. (2025). Multi-scale Multimodal Deep Learning Framework for Alzheimer’s Disease Diagnosis. Comput. Biol. Med..

[25] Cheng J., Wang H., Wei S., Mei J., Liu F., Zhang G. (2024). Alzheimer’s Disease Prediction Algorithm Based on De-correlation Constraint and Multi-modal Feature Interaction. Comput. Biol. Med..

[26] Leng Y., Cui W., Peng Y., Yan C., Cao Y., Yan Z., Chen S., Jiang X., Zheng J. (2023). Multimodal Cross Enhanced Fusion Network for Diagnosis of Alzheimer’s Disease and Subjective Memory Complaints. Comput. Biol. Med..

[27] Han K., Hu D., Zhao F., Liu T., Yang F., Li G. (2026). Incomplete Multi-modal Disentanglement Learning with Application to Alzheimer’s Disease Diagnosis. IEEE Trans. Med. Imaging.

[28] Zhang J., Gao Y., Gao Y., Munsell B.C., Shen D. (2016). Detecting Anatomical Landmarks for Fast Alzheimer’s Disease Diagnosis. IEEE Trans. Med. Imaging.

